# VNN1 promotes atherosclerosis progression in apoE^−/−^ mice fed a high-fat/high-cholesterol diet

**DOI:** 10.1194/jlr.M065565

**Published:** 2016-08

**Authors:** Yan-Wei Hu, Shao-Guo Wu, Jing-Jing Zhao, Xin Ma, Jing-Bo Lu, Jian-cheng Xiu, Yuan Zhang, Chuan Huang, Yu-Rong Qiu, Yan-Hua Sha, Ji-Juan Gao, Yan-Chao Wang, Shu-Fen Li, Jia-Yi Zhao, Lei Zheng, Qian Wang

**Affiliations:** Laboratory Medicine Center,* Nanfang Hospital, Southern Medical University, Guangzhou 510515, China; Department of Anesthesiology,† Nanfang Hospital, Southern Medical University, Guangzhou 510515, China; Department of Vascular Surgery,§ Nanfang Hospital, Southern Medical University, Guangzhou 510515, China; Department of Cardiology,** Nanfang Hospital, Southern Medical University, Guangzhou 510515, China

**Keywords:** apolipoprotein E, oxidized low density lipoprotein, vanin-1, inflammation

## Abstract

Accumulated evidence shows that vanin-1 (VNN1) plays a key part in glucose metabolism. We explored the effect of VNN1 on cholesterol metabolism, inflammation, apoptosis in vitro, and progression of atherosclerotic plaques in apoE^−/−^ mice. Oxidized LDL (Ox-LDL) significantly induced VNN1 expression through an ERK1/2/cyclooxygenase-2/PPARα signaling pathway. VNN1 significantly increased cellular cholesterol content and decreased apoAI and HDL-cholesterol (HDL-C)-mediated efflux by 25.16% and 23.13%, respectively, in THP-1 macrophage-derived foam cells (*P* < 0.05). In addition, VNN1 attenuated Ox-LDL-induced apoptosis through upregulation of expression of p53 by 59.15% and downregulation of expression of B-cell lymphoma-2 127.13% in THP-1 macrophage (*P* < 0.05). In vivo, apoE^−/−^ mice were divided randomly into two groups and transduced with lentivirus (LV)-Mock or LV-VNN1 for 12 weeks. VNN1-treated mice showed increased liver lipid content and plasma levels of TG (124.48%), LDL-cholesterol (119.64%), TNF-α (148.74%), interleukin (IL)-1β (131.81%), and IL-6 (156.51%), whereas plasma levels of HDL-C (25.75%) were decreased significantly (*P* < 0.05). Consistent with these data, development of atherosclerotic lesions was increased significantly upon infection of apoE^−/−^ mice with LV-VNN1. These observations suggest that VNN1 may be a promising therapeutic candidate against atherosclerosis.

Atherosclerosis is a multistep process in which apoptosis, lipids, inflammatory cells, and mediators orchestrate the formation and progression of plaques, which can lead to stroke and/or heart attack (1, 2). Epidemiologic studies have suggested that lipid abnormalities in blood are key risk factors for the development of atherosclerosis. The HDL-cholesterol (HDL-C) level is inversely correlated with the prevalence of coronary events and therefore has a cardioprotective effect (2, 3). It is now well accepted that atherosclerosis is not only a disorder of lipid metabolism, but also a chronic inflammatory disease. Inflammatory processes are involved at all stages of the atherosclerotic development, from lesion initiation to plaque rupture (4, 5). In addition, proapoptotic and antiapoptotic mechanisms contribute to the development and progression of atherosclerosis (6). For example, apoptosis of endothelial cells and smooth muscle cells is detrimental for plaque stability. Functional roles of macrophage apoptosis in atherosclerosis depend on the stages of plaque development. In advanced lesions, macrophage apoptosis increases, and advanced lesional macrophage apoptosis is associated with vulnerable plaques: plaque necrosis (7). However, in early atherosclerosis, macrophages play a proatherogenic role, and the turnover of macrophages by apoptosis limits lesion development (8). Therefore, factors that act to lower levels of cholesterol, limit inflammation, and selectively induce macrophage apoptosis in this setting may prove to be beneficial in reducing disease progression.

Vanin-1 (VNN1) is a glycosylphosphatidyl inositol-anchored pantetheinase that is highly expressed in the liver, gut, and kidney. It can catalyze the hydrolysis of pantetheine into cysteamine and pantothenic acid (vitamin B5) (9). Functional studies have suggested a role for VNN1 in oxidative stress, inflammation, and cell migration (10–13). For example, clinical investigations indicated that the levels of VNN1 were increased in the urine and blood of diabetic patients (14). VNN1 pantetheinase contributes to temporal and local tissue adaptation to needs and damage and participates in tissue tolerance to stress (15). Gensollen et al. (13) found that functional polymorphic positions in the VNN1 locus are direct targets for nuclear factors that might regulate the levels of VNN1 in colon and this would be linked to inflammatory bowel diseases susceptibility. These effects of VNN1 have attracted attention because evidence from in vitro systems and clinical studies suggests that it is a key activator for hepatic gluconeogenesis (14, 16). Importantly, the link between VNN1 and lipid metabolism has been revealed. For example, van Diepen et al. (17) showed that PPARα is the regulator of the expression and activity of VNN1 in the liver, which have key roles in the prevention of development of steatosis in response to fasting. A clinical study showed that VNN1 gene expression and the G-137T variant are associated with HDL-C levels in Mexican children, particularly in prepubertal girls (18). A sequencing effort yielded four SNPs in the VNN1 promoter region showed a high correlation with transcript abundance and HDL-C levels (19). However, these findings are descriptive, and the detailed mechanism through which VNN1 regulates lipid metabolism remains unknown.

In the present study, we demonstrated that oxidized LDL (Ox-LDL) could significantly induce VNN1 expression through an ERK1/2/cyclooxygenase-2 (COX-2)/PPARα signaling pathway. In addition, VNN1 promotes accumulation of cholesteryl esters by inhibiting expression of PPARγ and liver X receptor (LXR) α in THP-1 macrophage-derived foam cells Moreover, VNN1 attenuates Ox-LDL-induced apoptosis through upregulation of expression of p53 and downregulation of B-cell lymphoma-2 (BCL-2) in vascular smooth muscle cells (VSMCs). Consistent with this hypothesis, development of atherosclerotic lesions was increased significantly by infection of apoE^−/−^ mice with lentivirus (LV) encoding mouse VNN1 (LV-VNN1).

## MATERIALS AND METHODS

The present study conformed with the Declaration of Helsinki and was approved by the Review Board of Nanfang Hospital, Southern Medical University (Guangzhou, China). Written informed consent was obtained from all subjects. All procedures relating to the care and experimentation of animals were in accordance with the *Guide for the Care and Use of Laboratory Animals* (National Institutes of Health) and were approved by the Animal Experimental Committee of Nanfang Hospital.

### Materials

Human lipoproteins [Ox-LDL, acetylated-LDL (Ac-LDL), indocarbocyanine dye (Dil)-labeled Ox-LDL, and HDL] were obtained from Biomedical Technologies Inc. (Stoughton, MA). Transcriptor First Strand cDNA Synthesis kit and FastStart Universal SYBR Green Master (Rox) were obtained from Roche (Basel, Switzerland). Chemicals were obtained from Sigma-Aldrich (Saint Louis, MO) unless stated otherwise.

### Animals and diets

Male 8-week-old C57BL/6 mice and apoE^−/−^ mice with a C57BL/6 background were purchased from Vital River Laboratory Animal Technology Co. Ltd. (Beijing, China). They were housed five per cage at 25°C in a room on a 12 h light-dark cycle. To detect VNN1 gene expression levels, C57BL/6 mice were placed on either a chow diet (n = 5) or a high-fat diet (HFD; n = 5) for 3 weeks. The diet was a commercially prepared mouse food supplemented with 21% (w/w) butterfat, 0.15% (w/w) cholesterol, and 19.5% (w/w) casein (Beijing Keao Xieli Feed Co. Ltd., Beijing, China).To ascertain the effect of VNN1 on atherosclerosis, apoE^−/−^ mice were randomized into two groups of 20 and injected via the tail vein with control LV (LV-Mock) or LV-VNN1, respectively. Mice were fed an HFD for 12 weeks. At week 12, mice were inhalationally anesthetized with 2% isoflurane (Forene®, Abbott), 1 ml of blood was collected by cardiac puncture, mice were euthanized by cervical dislocation, and tissues were collected for further analysis. The adequacy of anesthesia was monitored by testing tactile stimulus response and forelimb or hind limb pedal withdrawal reflex, as well as continual observation of respiratory pattern, mucous membrane color, and responsiveness to manipulations throughout all the procedure.

### Macrophage isolation and culture

The primary peritoneal macrophages from mice were collected according to the method described by Lee et al. (20). Briefly, mice were injected intraperitoneally with 2 ml of sterile thioglycollate medium (Becton Dickinson, Franklin Lakes, NJ). Three days later, macrophages were collected by peritoneal lavage with cold DMEM. After centrifugation of the lavage, red blood cells were removed by lysis for 3 min in lysis buffer (150 mM NH_4_Cl, 1 mM KHCO_3_, and 0.1 mM Na_2_EDTA). After centrifugation, the cells were resuspended in DMEM supplemented with 1% antibiotic candantimycotic solution (Invitrogen, Carlsbad, CA) and 10% FCS, then incubated for 90 min in a humidified atmosphere of 5% CO_2_ at 37°C. Nonadherent cells were removed by washing, and the adherent cells were harvested and seeded for assays.

### Cell culture

Human monocytic THP-1 cells, HepG2 cells, Caco-2 cells, and human umbilical vein endothelial cells (HUVECs) were obtained from ATCC (Manassas, VA). THP-1 cells were cultured in RPMI 1640 medium containing 10% FCS and differentiated for 72 h with 100 nM PMA. Macrophages were transformed into foam cells by incubation in the presence or absence of 50 μg/ml of Ox-LDL in serum-free RPMI 1640 medium containing 0.3% BSA (BSA) for 48 h. HepG2 cells, Caco-2 cells, and HUVECs were grown in DMEM containing 10% FCS with streptomycin (100 mg/ml) and penicillin (100 U/ml). VSMCs were obtained from nonatherosclerotic areas of the aortas of organ donors of various ages (males and females aged from 5 years to 42 years). Cells were prepared from explants of tissue and were confirmed to be SM cells by positive staining with monoclonal antibodies against α-SM actin (A2547; Sigma-Aldrich). Cells were maintained in M199 containing 20% FCS and used between passages 3 and 10. All cells were incubated at 37°C in an atmosphere of 5% CO_2_. Cells were seeded in 6- or 12-well plates or 60 mm dishes and grown to 60–80% confluence before use.

### Apoptosis assay

Cells were harvested, and apoptosis quantification was undertaken by flow cytometry using a Fluorescent Activated Cell Sorting (FACScan) Flow Cytometer (Becton Dickinson Immuno­cytometry Systems, San Jose, CA) equipped with an argon-ion laser (488 nm). Binding to annexin V and propidium iodide (PI) was determined using an Apoptosis Detection kit according to the manufacturer’s instructions (Becton Dickinson Pharmingen, Oxford, UK). To distinguish between apoptosis and necrosis, cells were double-stained with annexin V (green fluorescence) and PI (red fluorescence). Briefly, cells (100,000 cells/sample) were washed twice in cold PBS and suspended in binding buffer containing fluorescein isothiocyanate-conjugated annexin V (10 μg/ml) and PI (10 μg/ml). The cell suspension was incubated in the dark for 15 min, and signals were acquired by the flow cytometer. A total of 10,000 events were analyzed for each sample with Cell Quest software (Becton Dickinson Biosciences, San Jose, CA).

### Cytokine assays and measurement of biochemical parameters in sera

Levels of human TNF-α, interleukin (IL)-1β, and IL-6 present in culture media (R and D Systems, Minneapolis, MN) and the serum concentrations of TNF-α, IL-1β (R and D Systems), IL-6 (R and D Systems), apolipoprotein (apoAI; Cusabio Biotech, Wuhan, China), and apoB100 (Cusabio Biotech Co. Ltd.) were measured by ELISA according to the manufacturer’s instructions. Plasma levels of total cholesterol (TC) and TGs were analyzed in an automated analyzer (AU5400; Beckman Coulter, Fullerton, CA). Lipoproteins were isolated by sequential ultracentrifugation from 60 μl of plasma at densities (d) of <1.006 g/ml (VLDL), 1.006 ≤ d ≤1.063 g/ml (intermediate density lipoprotein and LDL) and d >1.063 g/ml HDL) in an LE-80K Ultracentrifuge (Beckman Coulter). Cholesterol levels in lipoprotein fractions were determined by enzymatic means using a colorimetric method (AU5400; Beckman).

### RNA isolation and real-time quantitative PCR analyses

Total RNA from cultured cells was extracted using TRIzol Reagent (Invitrogen, Gaithersburg, MD) in accordance with the manufacturer’s instructions. Real-time quantitative PCR (qPCR) was undertaken on an ABI 7500 Fast Real-time PCR system with SYBR Green detection. Expression of glyceraldehyde 3-phosphate dehydrogenase was used as the internal control. Quantitative measurements were determined using the ^ΔΔ^Ct method. All samples were assayed in triplicate, and the mean value was considered for comparative analyses.

### Western blot analyses

Cells and tissues were harvested, and protein extracts prepared according to established methods. Extracts were separated by 10% sodium dodecyl sulfate–polyacrylamide gel electrophoresis and subjected to Western blot analyses using rabbit polyclonal antibodies against: LXRα, Niemann-Pick disease type C1 (NPC1), p53, BCL-2, steroid receptor RNA activator (SRA) 1, cluster of differentiation (CD) 36, sterol regulatory element-binding protein (SREBP) 1c, SREBP2, and VNN1 (Proteintech, Chicago, IL); intercellular adhesion molecule (ICAM)-1, vascular cell adhesion molecule (VCAM)-1, PPARγ, and ABCA1 (Abcam, Cambridge, MA); phosphorylated (p)-ERK1/2 (T202), COX-2, and PPARα (Bioworld Technology, Minneapolis, MN); ABCG1, scavenger receptor class B type I (SR-BI), LDL receptor (LDLR), HMG-CoA reductase (HMGCR), hydroxymethylglutaryl-CoA synthase, Niemann-Pick C1-like 1 protein (NPC1L1) (Epitomics, Burlingame, CA); and ABCG5 and β-actin antibody (Santa Cruz Biotechnology, Santa Cruz, CA). Proteins were visualized using a chemiluminescence method (ECL Plus Western Blot Detection System; Amersham Biosciences, Foster City, CA).

### Production and infection by LV

Human monocytic THP-1 cells, HepG2 cells, Caco-2 cells, HUVECs, and VSMCs were cultured. Packed empty LV vectors (LV-Mock) and LV-mediated VNN1 overexpression vector (LV-VNN1) were generated. Cells were transduced with LV stock at a multiplicity of infection of 100 (THP-1 cells and VSMCs) or 20 (HepG2 cells, Caco-2 cells, and HUVECs) transducing units per cell in the presence of 8 μg/ml of polybrene. Cells were washed with fresh complete media after 24 h of incubation.

### Transfection with siRNA mimics

THP-1 macrophages were transfected with 50 nM siRNAs against ERK1/2, COX-2, PPARα, and an irrelevant 21-nucleotide control siRNA (negative control) purchased from Ribo Targets (Cambridge, UK). Cells were transfected using Lipofectamine 2000 transfection reagent for 48 h according to the manufacturer’s instructions. All experimental control samples were treated with an equal concentration of a nontargeting control mimic sequence (negative controls). After 48 h of transfection, real-time RT-PCR and Western blot were conducted.

### Inhibitor treatment

ERK1/2 inhibitor PD98059 was purchased from Promega (Madison, WI). Cells were preincubated with 10 μM PD98059 for 1 h and then incubated with 50 μg/ml Ox-LDL for 48 h. COX-2 inhibitor NS-398 was purchased from Calbiochem (Merck, Bad Soden, Germany). Cells were preincubated with 1 μM NS-398 for 1 h and then incubated with 50 μg/ml Ox-LDL for 48 h. PPARα inhibitor GW6471 was purchased from Sigma (St. Louis, MO). Cells were preincubated with 0.5 μM GW6471 for 1 h, and then incubated with 50 μg/ml Ox-LDL for 48 h.

### Dil-Ox-LDL uptake assays

Fluorescent-tagged Dil-Ox-LDL was added to PMA-differentiated THP-1 cells for 1 h. Adherent cells were harvested and washed thrice with PBS. Analysis was done on a FACScalibur Flow Cytometer (Becton Dickinson) with Cell Quest Pro software (Becton Dickinson Biosciences).

### HPLC analyses of cellular levels of cholesterol

HPLC analyses were conducted as described previously (21). Sterol analyses were done using a HPLC system (Model 2790 controlled with Empower Pro software; Waters, Milford, MA). Absorbance at 216 nm was monitored. Data were analyzed with TotalChrom software from PerkinElmer (Waltham, MA).

### Cholesterol efflux from cells

THP-1 macrophages were cultured and labeled with 0.2 μCi/ml [^3^H]cholesterol. After 72 h, cells were washed with PBS and incubated overnight in RPMI 1640 medium containing 0.1% (w/v) BSA to allow equilibration of [^3^H]cholesterol in all cellular pools. Equilibrated [^3^H]cholesterol-labeled cells were washed with PBS and incubated in 2 ml of efflux medium containing RPMI 1640 medium and 0.1% BSA with 25 μg/ml of human plasma apoAI or 100 μg/ml of human plasma HDL for 12 h. A 150 μl sample of efflux medium was obtained at the times designated and passed through a 0.45 μm filter to remove floating cells. Monolayers were washed twice in PBS, and cellular lipids extracted with isopropanol. Medium and cell-associated levels of [^3^H]cholesterol were measured by liquid scintillation. Percent efflux was calculated by the equation: [total media counts/(total cellular counts + total media counts)] × 100%.

### In vivo RCT assay

An in vivo reverse cholesterol transport (RCT) assay was conducted as described previously (22). Bone marrow was isolated, and cells were plated overnight in DMEM supplemented with 10% FBS and 15% L-929 conditioned media. Nonadherent cells were removed and cultured for an additional 6 days to allow for macrophage differentiation. For RCT assays, bone marrow-derived macrophages (BMDMs) were washed twice and incubated with 37.5 μg/ml of Ac-LDL and 5 μCi/ml of [^3^H]cholesterol for 24 h. Cells were resuspended in ice-cold DMEM, and 3 × 10^6^ cells were injected subcutaneously into individually housed mice treated with LV-Mock or LV-VNN1 for 12 weeks, as described above. Before injection, an aliquot of cells was counted using liquid scintillation to measure baseline radioactivity. Blood was obtained by puncture of the saphenous vein 6, 12, and 24 h after BMDM injection and by cardiac puncture 48 h after euthanizing. An aliquot of plasma was used for liquid-scintillation counting immediately at each time point. The total feces collected from 0 h to 48 h was vacuum-dried and homogenized in 50% NaOH overnight, after which an aliquot was used for liquid-scintillation counting. At euthanizing, liver samples were collected and incubated with hexane/isopropanol (3:2) for 48 h and dried overnight. Lipids were resolubilized in liquid scintillation fluid and radioactivity counted. RCT to plasma, liver, and feces was calculated as a percentage of total radioactivity injected at baseline.

### En face plaque area

Immediately after euthanizing, aortas were excised and fixed in 10% buffered formalin for quantification of en face plaque areas. Briefly, after adventitial tissue was removed carefully, the aorta was opened along the longitudinal axis, stained with Oil Red O, and pinned on a blue wax surface. En face images were obtained using a stereomicroscope (SZX12; Olympus, Tokyo, Japan) equipped with a digital camera (Dxm1200; Nikon, Tokyo, Japan) and analyzed using Adobe Photoshop v7.0 and Scion Image software. The percentage of the luminal surface area stained by Oil Red O was determined.

### Quantification of atherosclerosis in the aortic sinus

The upper portion of the heart and proximal aorta were obtained, embedded in OCT compound (Fisher, Tustin, CA), and stored at −70°C. Serial 10 μm thick cryosections of the aorta, beginning at the aortic root, were collected at 400 μm. Sections were stained with Oil Red O. The area of positive staining for Oil Red O was calculated as a percentage of the total section area. An average size of lipid droplets was calculated utilizing ImagePro Plus software (Media Cybernetics, Rockville, MD) from five views per mouse.

### Liver staining using Oil Red O

To examine lipid deposition in the liver, lipid content was assessed in samples collected in OCT upon staining with Oil Red O. Briefly, liver cryosections were fixed for 10 min in 60% isopropanol, stained with 0.3% Oil Red O in 60% isopropanol for 30 min, and washed with 60% isopropanol. The distribution of Oil Red O staining across the liver was around portal and central vein. The area of positive staining for Oil Red O was calculated as a percentage of the total section area. An average size of lipid droplets was calculated utilizing ImagePro Plus software (Media Cybernetics) from five views per mouse.

### Immunohistochemical analyses

Each sample of frozen aortic root was sectioned (thickness, 5 μM) and fixed onto microscope slides. Sections of frozen tissue specimens were mounted on polylysine (Sigma-Aldrich)-coated slides, air-dried, and fixed with acetone. Immunohistochemical (IHC) staining was undertaken using rabbit polyclonal antibody to CD68 (1:100 dilution; Abcam), smooth muscle actin (SMA) (1:200 dilution; Dako), macrophage-3 (Mac-3) (1:150 dilution; Pharmingen), Ki67 (1:100 dilution; Novacastra Laboratories), and cleaved caspase 3 (1:100 dilution; Transduction Laboratories). Images were acquired and quantitated on a BX50 Microscope (Olympus) using Optimis v6.2 (Bedford, UK) and digitized using a color video camera (three-charge coupled device; JVC, Wayne, NJ). IHC evaluation for each protein was done by a semiquantitative method as described previously (23, 24). Images at ×400 magnification were acquired and processed in TIFF format using Image-Pro Plus® (Media Cybernetics). Slide analyses were done by measurement of positive pixel intensity using semiquantitative analysis tool-integrated optical density in the Image-Pro Plus v6.2 (Media Cybernetics). For the reading of staining of each antibody, a uniform setting for all the slides was applied. The area of positive staining for each protein was calculated as a percentage of total section area. The mean of these values was obtained for each sample and each study group and expressed as the mean ± SD.

### TUNEL staining

Apoptosis was quantified in paraffin-embedded aortic valve cross-sections using a terminal deoxynucleotidyl transferase-mediated deoxyuridine triphosphate nick-end labeling (TUNEL) Assay kit (In Situ Cell Death Detection Kit, POD; Roche Diagnostics, Mannheim, Germany) according to the manufacturer’s instructions. Dark-brown staining indicated positive TUNEL staining if slides were examined under a light microscope (Olympus BX53® Research Microscope; Olympus). Cells with clear nuclear labeling were defined as TUNEL-positive cells. Positive and negative control slides were processed simultaneously in each experiment. Total apoptosis was defined as the number of TUNEL-positive cells per 1,000 plaque cells.

### Double staining

Apoptotic or proliferating cells were identified as being either derived of macrophage or smooth muscle origin using a Vector Laboratories dual staining technique. The proliferative marker Ki-67 or cleaved caspase-3 were detected by using 3,3′-Diaminobenzidine (DAB) (Dako, Carpinteria, CA), with macrophages or VSMCs identified using Mac-3 or SMA counterstained blue using the Vector alkaline phosphatase substrate kit III (SK-5300).

### Statistical analyses

Data are the mean ± SD. Results were analyzed by Student’s *t*-test and one-way ANOVA followed by the Student-Newman-Keuls test using SPSS v13.0 (SPSS, Chicago, IL). *P* < 0.05 (two-tailed) was considered significant.

## RESULTS

### Ox-LDL induces VNN1 expression by the ERK1/2/COX-2/PPARα signaling pathway in THP-1 macrophages

**Fig. 1.**
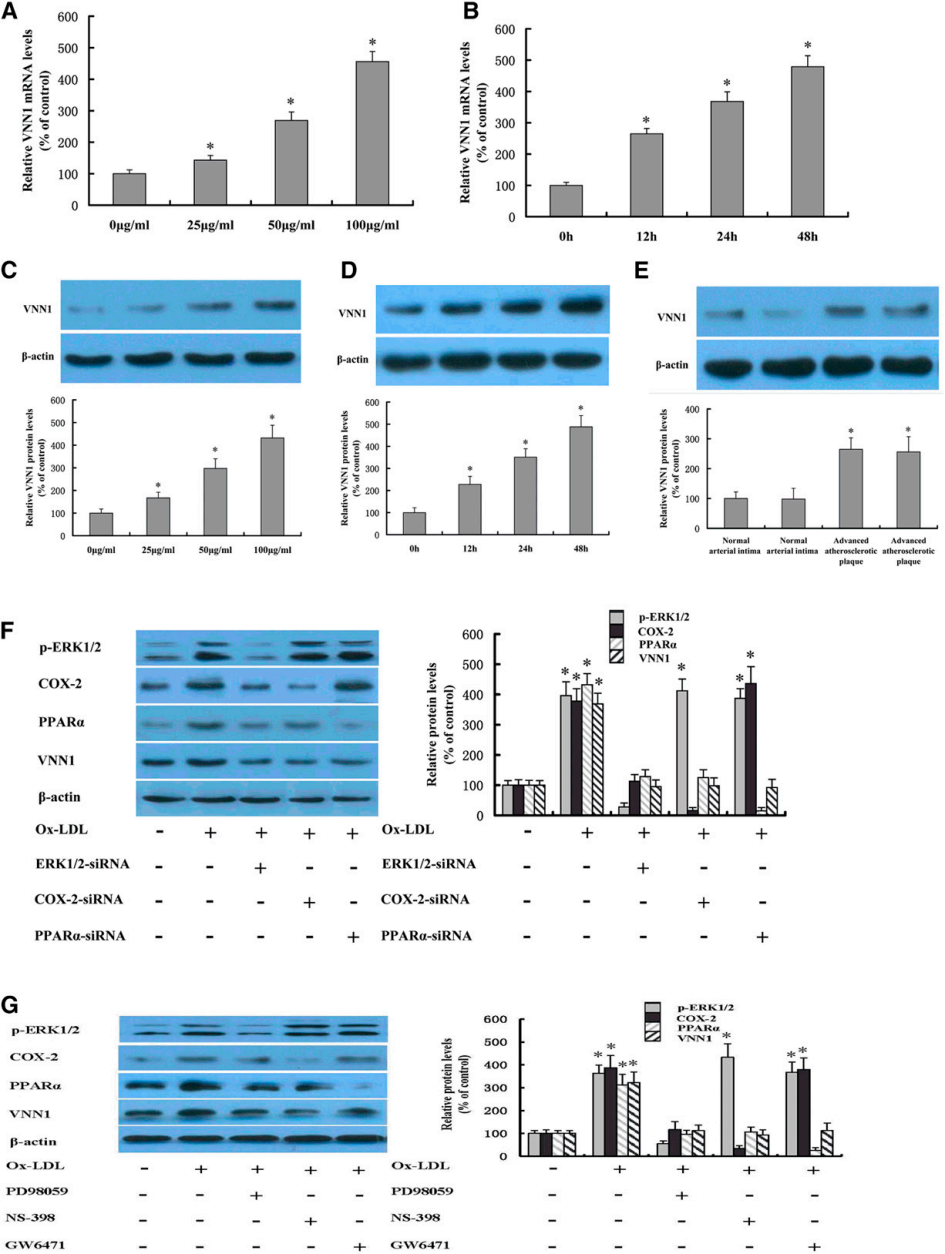
Effect and mechanism of Ox-LDL on VNN1 expression in THP-1 macrophages. A–D: Effects of Ox-LDL on VNN1 expression in THP-1 macrophages. A, C: THP-1 macrophages were treated with Ox-LDL at 0, 25, 50, and 100 μg/ml for 48 h, respectively. B, D: THP-1 macrophages were treated with 50 μg/ml of Ox-LDL for 0, 12, 24, and 48 h, respectively. Levels of VNN1 gene and protein were measured by real-time qPCR and Western blotting, respectively. E: Levels of VNN1 protein in human tissues of normal arterial intima (n = 5, 3 men; age 32.5 ± 7.8 years) and advanced atherosclerotic plaques (n = 5, 2 men; age 63.7 ± 8.9 years) were analyzed by Western blotting. F: THP-1 macrophages were transfected with control or ERK1/2 siRNA, COX-2 siRNA, or PPARα siRNA, and then incubated with 50 μg/ml Ox-LDL for 48 h. Protein expression was measured by Western blotting. G: THP-1 macrophages were transfected with control or ERK1/2 inhibitor PD98059, COX-2 inhibitor NS-398, or PPARα inhibitor GW6471, and then incubated with 50 μg/ml Ox-LDL for 48 h. Protein expression was measured by Western blotting. Data are the mean ± SD of five independent experiments, each done in triplicate. * *P* < 0.05 versus control.

To explore possible changes in RNA expression during foam cell formation, we undertook microarray analyses of THP-1 macrophages and THP-1 macrophages derived foam cells in the presence of Ox-LDL using the Arraystar probe data set, which comprises 24,420 coding transcripts (25, 26). Microarray results showed that expression of the VNN1 gene to be increased by 492% in THP-1 macrophage-derived foam cells compared with THP-1 macrophages (492 ± 181% vs. 100 ± 49%), suggesting that Ox-LDL affects the expression of VNN1 in THP-1 macrophages. Thus, we verified the effects of Ox-LDL on expression of VNN1 in THP-1 macrophages by RT-qPCR and Western blot analyses. VNN1 expression was obviously induced by Ox-LDL in a dose- and time-dependent manner (**Fig. 1A–D**). In addition, we found that protein expression of VNN1 was upregulated in advanced human atherosclerotic plaque tissues (type IV) in the abdominal aorta compared with normal human arterial intima tissues in the abdominal aorta (Fig. 1E). Recent studies have shown that Ox-LDL can upregulate expression of NPC1 through the ERK1/2/COX-2/PPARα signaling pathway in macrophages; hepatic VNN1 is under extremely sensitive regulation by PPARα: VNN1 activity in plasma can serve as a reflection of changes in PPARα activity in humans (17, 27). Therefore, our aim was to ascertain if Ox-LDL upregulates VNN1 expression through the ERK1/2/ COX-2/PPARα pathway in THP-1 macrophages.

ERK1/2 was not changed obviously along with the time of Ox-LDL incubation (data not shown). On the other hand, p-ERK1/2, expression of COX-2 protein, and expression of PPARα and VNN1 were significantly induced by Ox-LDL in THP-1 macrophages (Fig. 1F). Ox-LDL-induced upregulation of expression of COX-2, PPARα, and VNN1 was completely abolished by siRNA-mediated silencing of ERK1/2. Moreover, Ox-LDL-induced increase of expression of PPARα and VNN1 was abolished markedly by siRNA-mediated silencing of COX-2. Furthermore, upregulation of VNN1 expression could also be abolished by siRNA-mediated silencing of PPARα. Similarly, treatment with Ox-LDL could increase the expression of p-ERK1/2, COX-2, PPARα, and VNN1. Ox-LDL-induced upregulation of expression of COX-2, PPARα, and VNN1 was completely abolished by ERK1/2 inhibitor PD98059. Moreover, the increase of expression of PPARα and VNN1 induced by Ox-LDL was abolished markedly by COX-2 inhibitor NS-398. Furthermore, upregulation of VNN1 expression could also be abolished by PPARα inhibitor GW6471 (Fig. 1G). These data suggest that Ox-LDL can induce VNN1 expression through an ERK1/2/COX-2/PPARα signaling pathway in THP-1 macrophages.

### VNN1 promotes accumulation of cholesteryl esters by inhibiting expression of PPARγ and LXRα in THP-1 macrophage-derived foam cells

**Fig. 2.**
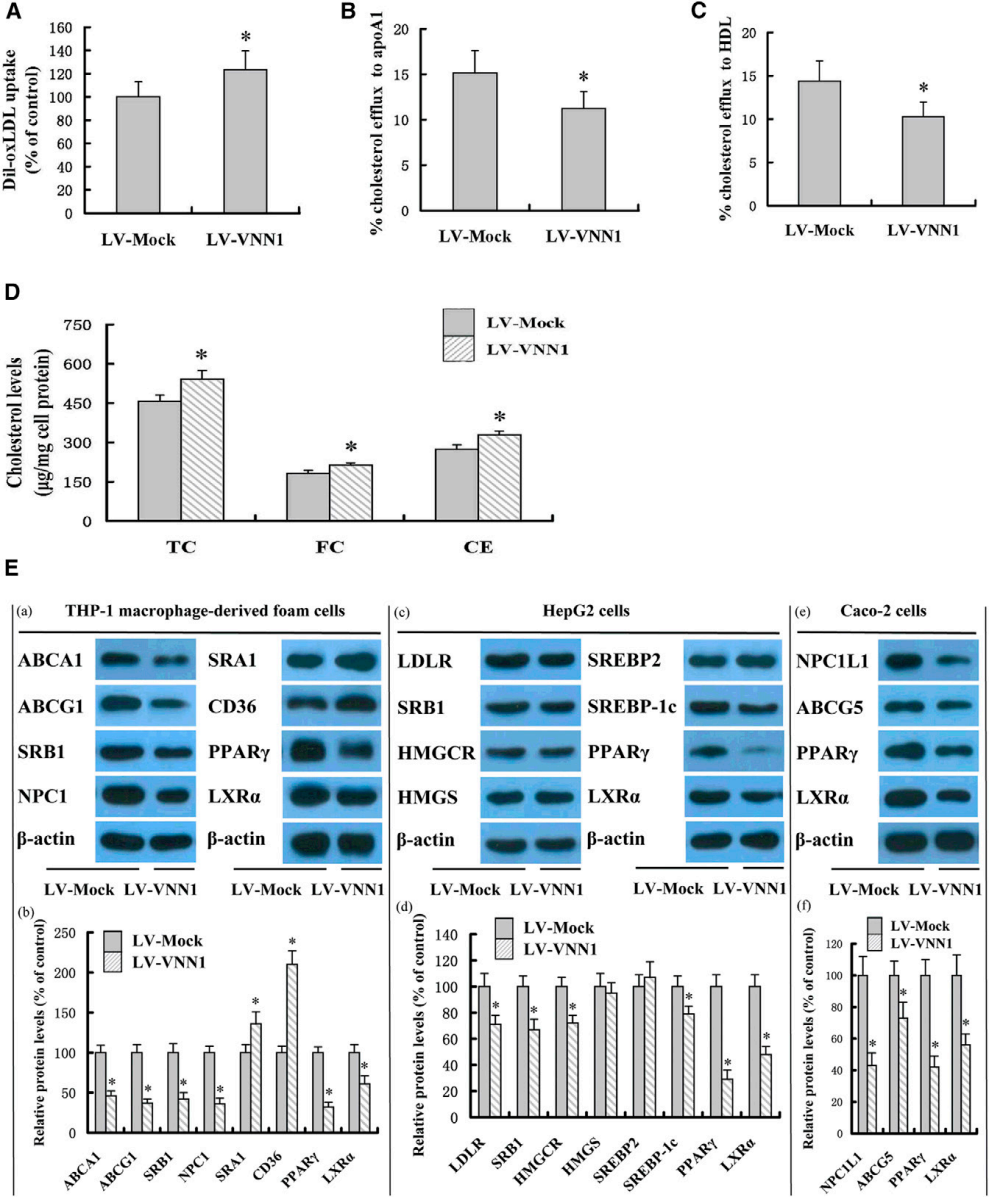
Effect of VNN1 on lipid loading, lipid content, and cholesterol efflux. THP-1 macrophages were transduced with LV-Mock or LV-VNN1. A: Then cells were incubated with 5 μg/ml of Dil-labeled Ox-LDL for 1 h and uptake of Dil-labeled Ox-LDL analyzed by flow cytometry. B, C: Cells were labeled with 0.2 μCi/ml [^3^H]cholesterol and cholesterol-loaded using 50 μg/ml Ox-LDL. The percentage of cholesterol efflux to apoAI (B) and HDL (C) was analyzed with a liquid scintillation counting assay. D: HPLC was done to determine the TC, free cholesterol (FC), and cholesteryl ester (CE) content in cells. E: Protein levels were measured by Western blot analyses. Data are the mean ± SD from five independent experiments, each carried out in triplicate. * *P* < 0.05 versus control group.

First, we investigated the role of VNN1 on lipid loading in THP-1 macrophages via flow cytometry. Dil-Ox-LDL uptake was increased by LV-mediated overexpression of VNN1 (**Fig. 2A**). Next, we examined the effect of VNN1 on cholesterol content and efflux in THP-1 macrophage-derived foam cells. Cellular cholesterol content (Fig. 2D) was increased, whereas cholesterol efflux to apoAI and HDL (Fig. 2B, C) was decreased in cells treated with LV-mediated overexpression of VNN1 in THP-1 macrophages. Subsequently, we explored the mechanisms underlying the altered cellular lipid profile by VNN1 in THP-1 macrophage-derived foam cells, HepG2 cells, and Caco-2 cells. Protein levels of ABCA1, ABCG1, SR-BI, and NPC1 were downregulated, whereas protein levels of SRA1 and CD36 were upregulated by LV-mediated overexpression of VNN1 in THP-1 macrophage-derived foam cells (Fig. 2E). In addition, protein levels of LDLR, SR-BI, HMGCR, and SREBP1c were downregulated by VNN1 overexpression in HepG2 cells. Moreover, protein levels of NPC1L1 and ABCG5 were downregulated by VNN1 overexpression in Caco-2 cells. Furthermore, we found that protein levels of PPARγ and LXRα were downregulated significantly by VNN1 overexpression in THP-1 macrophage-derived foam cells, HepG2 cells, and Caco-2 cells. Previous studies from our research team and others have showed that expression of ABCA1, ABCG1, SR-BI, NPC1, LDLR, HMGCR, SREBP1c, NPC1L1, and ABCG5 can be upregulated, whereas that of SRA1 and CD36 can be downregulated by PPARγ and LXRα (4, 28). Taken together, VNN1 could increase accumulation of cholesteryl esters through inhibition of expression of PPARγ and LXRα in THP-1 macrophage-derived foam cells.

### VNN1 attenuates Ox-LDL-induced apoptosis through upregulation of expression of p53 and downregulation of BCL-2 in THP-1 macrophage

**Fig. 3.**
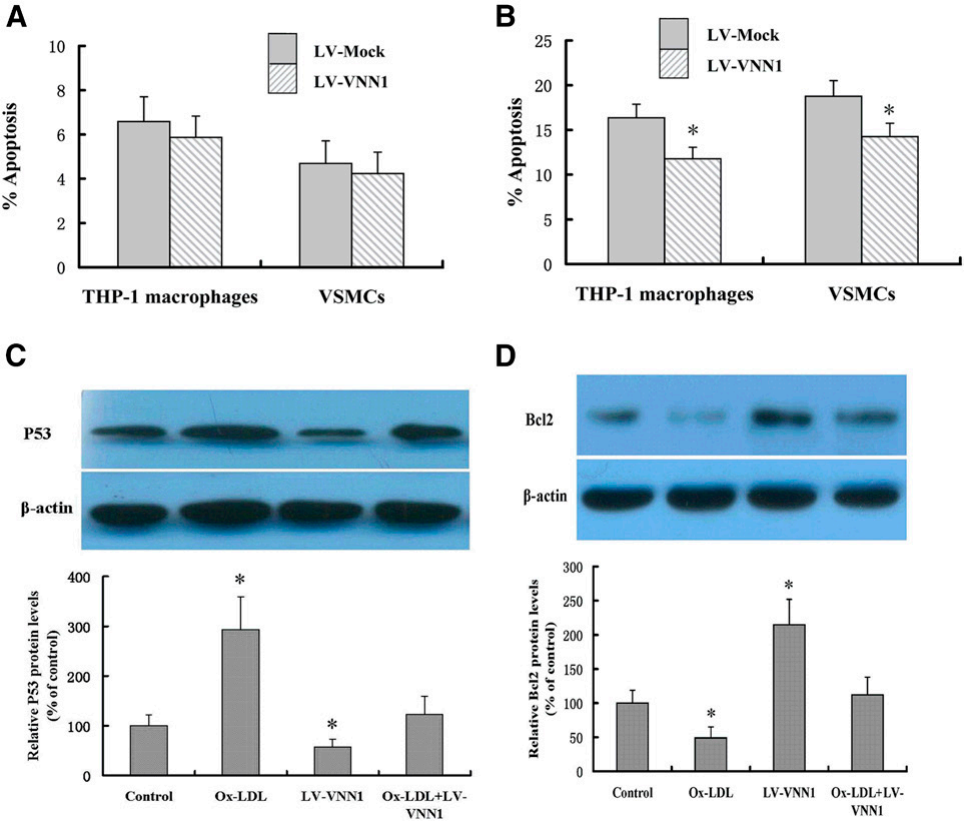
Effect and mechanism of VNN1 on apoptosis. A: THP-1 macrophages and VSMCs were transduced with LV-Mock or LV-VNN1, respectively. B: THP-1 macrophages and VSMCs were transduced with LV-Mock or LV-VNN1 and then treated with 100 μg/ml of Ox-LDL for 24 h, respectively. A, B: The proportion of apoptotic cells was assessed by flow cytometry. C, D: THP-1 macrophage were transduced with LV-Mock or LV-VNN1 and then treated with or without 100 μg/ml of Ox-LDL for 24 h as indicated. Protein levels were measured by Western blot analyses. Data are the mean ± SD from five independent experiments, each undertaken in triplicate. * *P* < 0.05 versus control group.

Apoptosis plays a key part in the development and progression of atherosclerosis. The effect of apoptosis in atherosclerosis is dependent upon the stage of the plaque, localization, and cell types involved. Macrophages and smooth muscle cells undergo apoptosis in atherosclerotic plaques (7). Here, we investigated the effects of VNN1 on apoptosis of THP-1 macrophages and VSMCs. We found that treatment with the LV-VNN1 vector did not change the percentage of apoptotic THP-1 macrophages and VSMCs (**Fig. 3A**), but significantly attenuated the Ox-LDL-induced percentage of apoptotic cells in comparison with treatment with the LV-Mock vector in THP-1 macrophages (28.0%) and VSMCs (24.0%), respectively (Fig. 3B). Next, we explored the mechanisms underlying the altered percentage of apoptotic THP-1 macrophages after treatment with overexpressed VNN1. We found that VNN1 overexpression markedly inhibited levels of p53 protein. The upregulation of p53 expression induced by Ox-LDL was abolished by treatment with overexpressed VNN1 (Fig. 3C). VNN1 overexpression also significantly increased levels of BCL-2 protein, and the downregulation of BCL-2 expression induced by Ox-LDL was markedly compensated by VNN1 overexpression (Fig. 3D). These data indicated that VNN1 may attenuate Ox-LDL-induced apoptosis through upregulation of expression of p53 and downregulation of BCL-2 expression in THP-1 macrophages.

### VNN1 treatment decreases reverse cholesterol transport and increases circulating cytokine levels in vivo

**Fig. 4.**
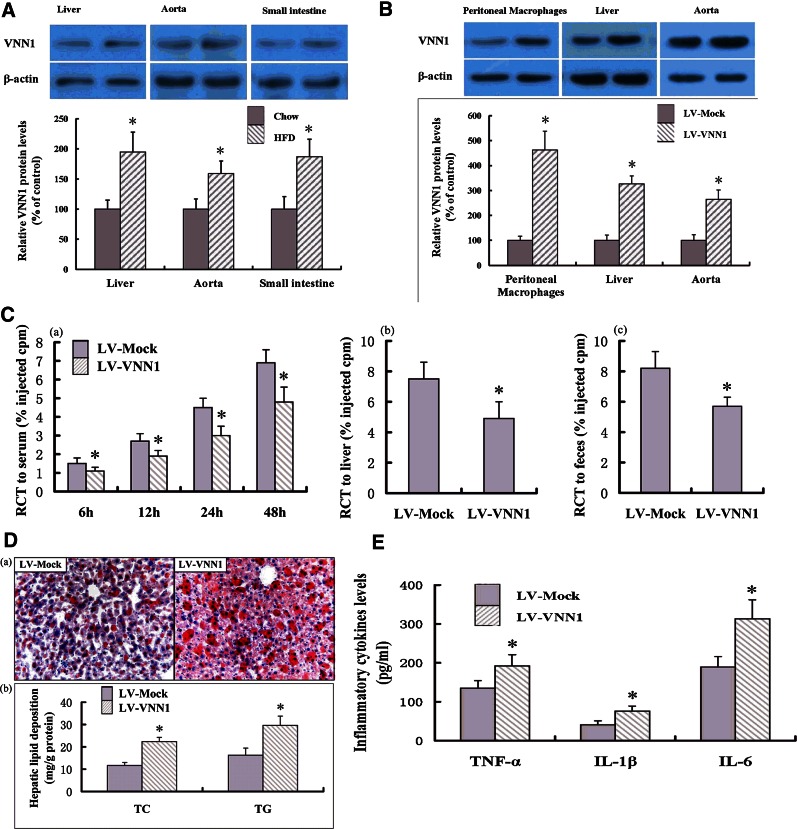
Effect of VNN1 on RCT and lipid deposition in the liver. A: C57BL/6 mice were treated with an HFD or a chow diet. VNN1 protein expression levels in various tissues were measured by Western blot. B: The expression levels of VNN1 in the LV-treated mice were measured by Western blot. C: After 12 weeks of the treatment indicated, apoE^−/−^ mice were injected subcutaneously with [^3^H]cholesterol-labeled Ac-LDL-loaded BMDMs. Data are the percentage of the [^3^H]cholesterol tracer relative to that of total counts per minute tracer injected ± SD; n = 10, * *P* < 0.05 versus control group. (a) Time course of [^3^H]cholesterol distribution in plasma. (b) Hepatic levels of [^3^H]cholesterol tracer after 48 h. (c) Fecal levels of [^3^H]cholesterol tracer. Feces were collected continuously from 0 h to 48 h postinjection. D: (a) Liver cryosections were stained with Oil Red O. Original magnification: ×100. (b) Hepatic content of TG and TC in apoE^−/−^ mice was assessed by enzymatic analyses. n = 10, * *P* < 0.05 versus control group. E: THP-1 macrophage-derived foam cells were treated with LV-Mock or LV-VNN1, respectively, and then levels of inflammatory cytokines in the medium measured with ELISA. Data are the mean ± SD from five independent experiments, each done in triplicate. * *P* < 0.05 versus control group.

We first analyzed VNN1 protein expression levels in various tissues of C57BL/6 mice with an HFD or a chow diet by Western blot. As shown in **Fig. 4A**, VNN1 expression was significantly upregulated in liver, aorta, and small intestine tissues of apoE^−/−^ mice when fed with an HFD. Moreover, the expression levels of VNN1 in the LV-treated mice were also analyzed. Treatment with LV encoding mouse VNN1 (LV-VNN1) injection significantly increased the expression level of VNN1 in peritoneal macrophages, liver, and aorta tissues compared with control group (LV-Mock) (Fig. 4B).

Because of the key role of VNN1 in the metabolism of cholesterol and lipids, we examined further the effect of VNN1 on terminal plasma lipid levels in apoE^−/−^ mice fed an HFD. VNN1 overexpression led to a moderate increase in plasma levels of TG (24.1%) and LDL-cholesterol (LDL-C; 19.6%) (**Table 1**). Concomitantly, plasma levels of HDL-C (25.8%) showed a moderate decrease in the VNN1 group compared with the control group. To ascertain if lower HDL-C levels in response to LV-VNN1 treatment could inhibit cholesterol transport from peripheral cells to the liver for further excretion into bile and feces, we undertook an in vivo RCT assay to trace [^3^H]cholesterol from cholesterol-loaded macrophages ex vivo (Fig. 4A). LV-VNN1-treated mice injected subcutaneously with cholesterol-loaded/[^3^H]cholesterol-labeled BMDMs showed a 30.4% decrease in [^3^H]cholesterol content in plasma over 48 h compared with that of LV-Mock-treated mice. Furthermore, LV-VNN1-treated mice showed a 34.7% decrease in the delivery of a ^3^H tracer to the liver and a 30.5% decrease in ^3^H sterols excreted into the feces. These results suggested that enhanced expression of VNN1 could not only decrease circulating HDL-C levels and increase circulating levels of TG and LDL-C, but also inhibit the RCT pathway in vivo.

**TABLE 1.** Effect of VNN1 on serum levels of lipids and lipoproteins in apoE^−/−^ mice (n = 10)

	LV-Mock	LV-VNN1
TG (mM)	1.43 ± 0.19	1.78 ± 0.22*^a^*
TC (mM)	32.79 ± 3.19	34.43 ± 3.63
HDL-C (mM)	7.96 ± 1.61	5.91 ± 1.49*^a^*
LDL-C (mM)	19.60 ± 2.96	23.45 ± 2.76*^a^*
VLDL-C (mM)	5.23 ± 1.13	5.07 ± 1.32
ApoAI (g/l)	0.06 ± 0.01	0.06 ± 0.02
ApoB (g/l)	0.15 ± 0.03	0.16 ± 0.03

Data are the mean ± SD.

a *P* < 0.05 versus LV-Mock group.

Because of the key role of the liver in determining plasma levels of lipoproteins, several therapeutic strategies designed to modulate hepatic lipid metabolism have been developed to reduce the susceptibility to atherosclerosis. Therefore, we next analyzed the effect of VNN1 on the lipid content of the liver in apoE^−/−^ mice by Oil Red O staining. Representative images of randomly selected sections of the liver stained for Oil Red O in the control group and VNN1 group are shown in Fig. 4B. Staining by Oil Red O revealed that liver lipid content was increased in LV-VNN1-treated animals compared with controls. Assessment of the effect of VNN1 on the content of TG and TC by enzymatic analyses showed a moderate increase of TG (32.4%) and TC (44.0%) in the LV-VNN1 group (Fig. 4B).

Atherosclerosis is a complex inflammatory disease, with macrophage foam cells being the major cell type involved in inflammation. Therefore, we investigated the effects of VNN1 on expression of inflammatory factors in THP-1 macrophage-derived foam cells. Notably, after treatment with overexpressed VNN1, secretion of the inflammatory cytokines TNF-α (42.2%), IL-1β (85.4%), and IL-6 (65.6%) into the culture medium was increased markedly (Fig. 4C). Moreover, to ascertain if VNN1-mediated changes in cellular expression of proinflammatory genes could result in corresponding changes in expression of inflammatory cytokines in plasma, we conducted a series of ELISAs (**Table 2**). Consistent with the data of expression of inflammatory genes in our in vitro experiment, infection with LV-VNN1 increased the plasma concentrations of TNF-α, IL-1β, and IL-6 by 32.8%, 31.8%, and 56.5%, respectively.

**TABLE 2.** Effect of VNN1 on serum levels of cytokines in apoE^−/−^ mice (n = 10)

	TNF-α (pg/ml)	IL-1β (pg/ml)	IL-6 (pg/ml)
LV-Mock	12.27 ± 1.69	14.65 ± 1.68	87.23 ± 6.53
LV-VNN1	18.25 ± 2.36*^a^*	19.31 ± 2.75*^a^*	136.52 ± 7.78*^a^*

Data are the mean ± SD.

a *P* < 0.05 versus LV-Mock group.

### VNN1 treatment promotes formation of atherosclerosis plaques in apoE^−/−^ mice

**Fig. 5.**
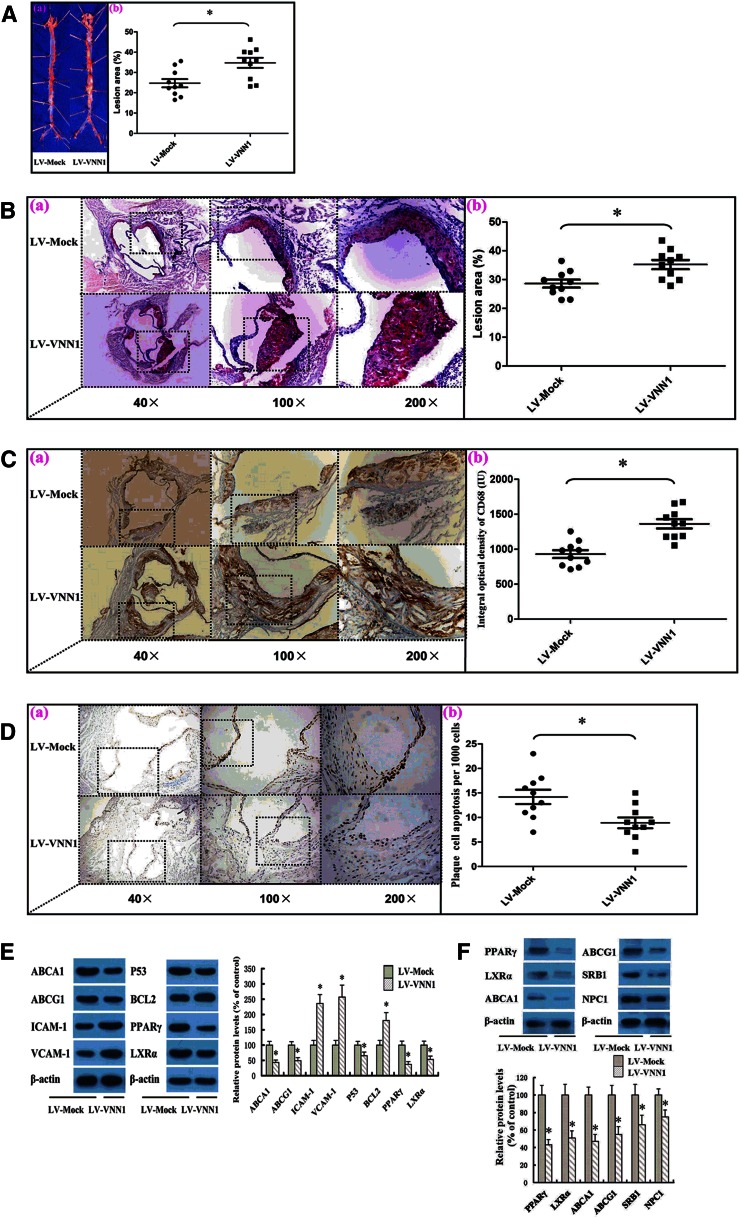
Effect of VNN1 on the initiation and development of atherosclerosis in apoE^−/−^ mice. A: (a) Representative staining of en face aorta with Oil Red O. (b) En face lesions were analyzed in apoE^−/−^ mice. B: (a) Representative staining of aortic valves with Oil Red O. (b) Total lesions in the aortic valves of apoE^−/−^ mice were analyzed. C: (a) Cryosections of aortic valves from apoE^−/−^ mice were stained by IHC means for the macrophage marker CD68. (b) The integral optical density of CD68 in aortic-valve cryosections from apoE^−/−^ mice was analyzed. D: (a) Representative image of aortic valves with TUNEL staining. (b) Total apoptosis in the aortic valves of apoE^−/−^ mice was analyzed. E: Levels of protein expression in the aortic tissues of apoE^−/−^ mice were analyzed by Western blotting. F: Levels of protein expression in peritoneal macrophages derived from apoE^−/−^ mice were analyzed by Western blot (n = 5 mice/group). Data are the mean ± SD; n = 10. All experiments were done in triplicate, except as indicated. * *P* < 0.05 versus control group.

To further understand the effects of VNN1 on atherogenesis in apoE^−/−^ mice, atherosclerotic lesions were evaluated by aortic-valve section and en face analyses (**Fig. 5A, B**). Mice receiving LV-mediated overexpressed VNN1 showed an increase in average lesion area compared with controls by en face and aortic-valve section analyses. Quantification of Oil Red O-stained lesions in en face preparations of aortas revealed that treatment with LV-VNN1 resulted in a significant (40.5%) increase in lesion area compared with controls. To confirm the negative effects of VNN1 on atherosclerosis, Oil Red O-stained aortic-valve sections were quantified and showed a significant (23.1%) increase in lesion area in apoE^−/−^ mice treated with LV-VNN1 compared with control.

Subsequently, the composition of the observed plaques was determined by IHC staining. The number of CD68-positive cells in plaques of the LV-VNN1-treated group was more than that in the control group (Fig. 5C). LV-VNN1 treatment resulted in a significant increase in MAC-positive lesions area (**Table 3**). In addition, because overexpression of VNN1 could attenuate the percentage of Ox-LDL-induced apoptotic cells in vitro, we quantified total apoptosis in atherosclerotic plaques from apoE^−/−^ mice. Lesions from LV-VNN1-treated mice showed a significant decrease in apoptosis compared with control mice (Fig. 5D and Table 3). Moreover, we found that LV-VNN1 treatment reduced cleaved caspase 3-positive cells expressing MAC-3 by 38.6% compared with LV-Mock treatment, with no significant reduction in cleaved caspase 3-positive cells expressing SMA (Table 3).

**TABLE 3.** Histological analysis of control ApoE^−/−^ mice treated with LV-VNN1 (n = 10)

	LV-Mock	LV-VNN1
α-SMA-positive area, %	3.65 ± 0.97	3.59 ± 1.06
MAC-positive area, %	35.76 ± 4.89	47.57 ± 9.63*^a^*
Ki67-positive cells, %	1.53 ± 0.42	1.51 ± 0.47
Cleaved caspase 3-positive cells, %	0.93 ± 0.25	0.72 ± 0.29*^a^*
Ki67-positive cells expressing SMA, %	3.21 ± 0.23	3.28 ± 0.36
Ki67-positive cells expressing MAC-3, %	7.69 ± 0.78	7.71 ± 0.86
Cleaved caspase 3-positive cells expressing SMA, %	2.78 ± 0.62	2.69 ± 0.73
Cleaved caspase 3-positive cells expressing MAC-3, %	8.62 ± 1.21	5.29 ± 0.96*^a^*

Data are the mean ± SD.

a *P* < 0.05 versus LV-Mock group.

To explore the mechanisms whereby VNN1 treatment promoted the progression and stabilization of plaques, changes in protein expression of the inflammatory factors, apoptosis regulatory factors, and molecules involved in cellular efflux of cholesterol were investigated in the aorta of apoE^−/−^ mice (Fig. 5E). In LV-VNN1-treated apoE^−/−^ mice, protein expression of ABCA1, ABCG1, P53, PPARγ, and LXRα was decreased markedly, but protein expression of ICAM-1, VCAM-1, and BCL-2 increased, at 12 weeks. Moreover, we isolated and cultured primary peritoneal macrophages to further confirm the impact of VNN1 on PPARγ target genes and activation of RCT. As shown in (Fig. 5F), protein levels of PPARγ, LXRα, ABCA1, ABCG1, SR-BI, and NPC1 were downregulated by LV-mediated overexpression of VNN1 in macrophages derived from apoE^−/−^ mice. These results further reinforce the contribution of VNN1-mediated PPARγ regulation in macrophages.

## DISCUSSION

**Fig. 6.**
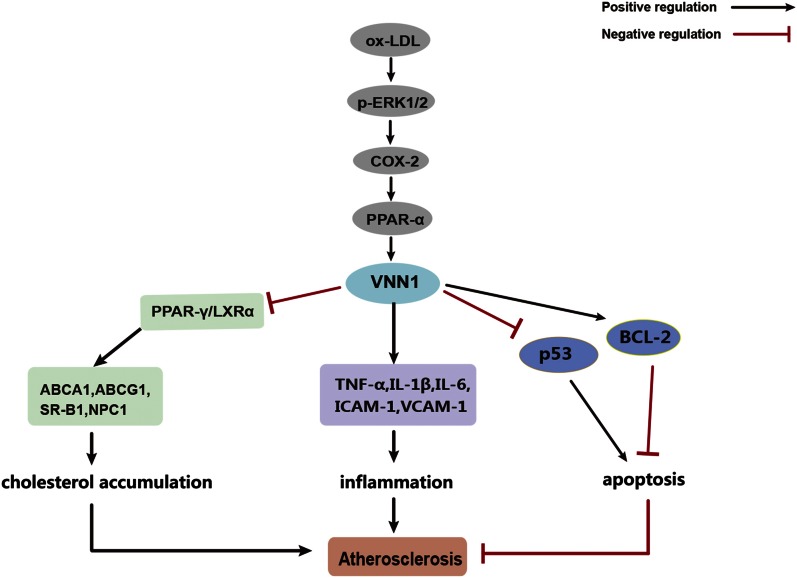
VNN1 expression and function can be regulated by Ox-LDL to promote atherosclerosis. These harmful effects are accompanied by decrease of cholesterol efflux, promotion of inflammatory molecule expression, and decrease of macrophage apoptosis, thus promoting atherosclerotic plaque formation. Ox-LDL upregulates VNN1 expression through ERK1/2/COX-2/PPARα signaling pathway. Then, VNN1 could inhibit cellular cholesterol efflux through reducing expression of ABCA1, ABCG1, SR-BI, and NPC1 by inhibiting expression of PPARγ and LXRα. In addition, VNN1 could promote inflammation through inducing expression of TNF-α, IL-1β, IL-6, ICAM-1, and VCAM-1. Moreover, VNN1 might reduce macrophage apoptosis through inhibiting P53 expression and inducing BCL-2 expression.

Atherosclerosis and the subsequent cardiovascular complications, such as stroke, myocardial infarction, and ischemic heart failure, is a major cause of death in the Western world. It is a chronic inflammatory process that is characterized by the formation of plaques consisting of foam cells, immune cells, vascular endothelial cells, smooth muscle cells, the extracellular matrix, platelets, and a lipid-rich core with extensive necrosis and fibrosis of surrounding tissues (29). It is believed that atherogenesis involves highly specific biochemical and molecular responses with constant interactions between various cellular players (30). In this study, we demonstrated that Ox-LDL significantly induced VNN1 expression through the ERK1/2/COX-2/PPARα signaling pathway. In addition, VNN1 could promote accumulation of cholesteryl esters by inhibiting expression of PPARγ and LXRα in THP-1 macrophage-derived foam cells. Furthermore, VNN1 could increase expression of inflammatory factors and attenuate Ox-LDL-induced apoptosis in vitro. Consistent with these data, VNN1 might increase plasma levels of TG and LDL-C, decrease plasma levels of HDL-C, inhibit reverse cholesterol transport, and promote circulating cytokine levels, leading to a significant promotion of formation of atherosclerotic plaques in apoE^−/−^ mice fed a high-fat/high-cholesterol diet (Fig. 6). These results suggest that VNN1 is a promising therapeutic target for atherosclerosis.

Formation of macrophage foam cells in the intima is a major hallmark of early stage atherosclerotic lesions. Uncontrolled uptake of Ox-LDL, impaired release of cholesterol, and excessive cholesteryl esterification could result in accumulation of cholesteryl esters stored as cytoplasmic lipid droplets and subsequently trigger the formation of foam cells (31). Control of the homeostasis of cholesterol in macrophages is of critical importance in the pathogenesis of atherosclerosis. We observed that VNN1 exerted an atherogenic effect by promoting Ox-LDL uptake, inhibiting cholesterol efflux to apoAI and HDL, and increasing cellular cholesterol content in THP-1 macrophage-derived foam cells. We also found that protein levels of ABCA1, ABCG1, SR-BI, and NPC1 were downregulated by VNN1 overexpression in THP-1 macrophage-derived foam cells. Furthermore, we found that protein levels of PPARγ and LXRα were downregulated significantly by VNN1 overexpression in THP-1 macrophage-derived foam cells. Previous studies from our research team and others have shown that expression of ABCA1, ABCG1, SR-BI, and NPC1 can be upregulated by PPARγ and LXRα in THP-1 macrophage-derived foam cells (4, 28). Taken together, VNN1 could increase accumulation of cholesteryl esters through inhibition of expression of PPARγ and LXRα in THP-1 macrophage-derived foam cells. Furthermore, we undertook an in vivo RCT assay that measured the integrated rate of movement of [^3^H]cholesterol from macrophages to the serum, liver, and feces. We showed that VNN1 could decrease the flux of cholesterol from cholesterol-loaded macrophages to all three of these compartments. The liver has been proposed to be a “metabolic center” for cholesterol and lipoproteins, so we analyzed the effects of VNN1 on hepatic lipid content. We found that the content of TG and TC was increased significantly in the liver of apoE^−/−^ mice treated with LV-VNN1. Consistent with these observations, plasma levels of TG and LDL-C were increased, whereas plasma levels of HDL-C were decreased in LV-VNN1-treated apoE^−/−^ mice fed an HFD. These results provide strong evidence to support the notion that VNN1 contributes to an atherogenic effect through increased uptake of, as well as inhibited trafficking and efflux of, cholesterol, thereby increasing lipid content, promoting foam-cell formation, and attenuating the rate of RCT. Moreover, previous study has found that VNN1 encodes a glycosylphosphatidylinositol-linked membrane-associated pantetheinase that catalyzes the hydrolysis of pantetheine into pantothenic acid (vitamin B5) (15). Multiples lines of experimentation clearly demonstrated that vnn1 is a direct PPARα gene (17, 32, 33). Our study also demonstrated that Ox-LDL significantly induced VNN1 expression through the ERK1/2/COX-2/PPARα signaling pathway, which reminds us that this pathway may also be involved in the regulation of the metabolism of pantothenic acid. Further work is needed before a definitive conclusion on this matter can be drawn.

In recent years, scholars have found that inflammation is a major driving force in the development of atherosclerotic lesions. It is well established that from early lesions to formation of vulnerable plaques, numerous cellular and molecular inflammatory components participate in disease (31). Monocytes migrate from the circulation into the intima of the arterial wall, where they differentiate into macrophages that then take up modified lipoproteins, thereby transforming into foam cells (34, 35). Monocyte-derived macrophages are abundantly present at all stages of the disease process. Macrophage-associated cytokines such as TNF-α, IL-1β, and IL-6 have emerged as key factors in the pathogenesis of atherosclerosis (34, 36, 37). In the present study, we showed that VNN1 significantly upregulated expression of TNF-α, IL-1β, and IL-6 in THP-1 macrophage-derived foam cells. To further investigate the mechanisms whereby VNN1 treatment promoted the progression and stabilization of plaques, changes in levels of inflammatory molecules were explored in apoE^−/−^ mice fed an HFD. We found that plasma levels of TNF-α, IL-1β, and IL-6 were increased markedly in VNN1-overexpressing apoE^−/−^ mice. Our results suggest that VNN1-induced expression of inflammatory molecules can promote the development of atherosclerotic lesions and thus have a negative influence on disease outcome.

Apoptosis plays a key part in the pathogenesis of various cardiovascular diseases. Apoptosis, as identified by morphologic and molecular changes, is increased in aged vascular cells and is also increased in VSMCs and other cells in atherosclerotic plaques. The greatest amount of cell death in plaques is within the macrophage-rich necrotic core, whereas apoptosis occurs in the macrophages and VSMCs of the plaque (29). We investigated the effects of VNN1 on apoptosis of THP-1 macrophages and VSMCs. We showed that overexpression of VNN1 could attenuate the percentage of Ox-LDL-induced apoptotic THP-1 macrophages and VSMCs in comparison with controls, respectively. In addition, we found that lesions from LV-VNN1-treated mice showed a decrease in macrophage apoptosis when compared with control mice. Consistent with these data, protein expression of p53 was decreased markedly, whereas protein expression of BCL-2 was increased in LV-VNN1-treated apoE^−/−^ mice as compared with controls. Furthermore, we found that VNN1 overexpression could markedly inhibit levels of p53 protein and compensated for Ox-LDL-induced upregulation of expression of p53 protein THP-1 macrophage. VNN1 overexpression could also significantly increase levels of BCL-2 protein and compensate for Ox-LDL-induced downregulation of expression of BCL-2 protein THP-1 macrophage. It is interesting that p53 and bcl-2 were markedly regulated by VNN1 overexpression while the impacts of VNN1 overexpression on the Ox-LDL-induced cell apoptosis are of modest amplitude. Perhaps there still exist other pathways that regulate cell apoptosis and compensate the downregulation of apoptosis by VNN1. In short, these data suggest that VNN1 may attenuate Ox-LDL-induced apoptosis through upregulation of expression of p53 and downregulation of expression of BCL-2. Thus, inhibition of VNN1 expression to induce apoptosis of cells in early lesions could be important for reducing the number and size of cells in atherosclerotic lesions and promote the formation of more stable plaques.

Several limitations exist in our study. In our present study, lipoproteins and lipid levels were measured by ELISA or automated analyzer or isolated by sequential ultracentrifugation. It is more rigorous to perform further analyses of the plasma lipoprotein by fast protein liquid chromatography and agarose gel electrophoresis to confirm our findings. Second, LV was injected via tail vein to overexpress VNN1 in vivo study. It is still not clear whether endogenous VNN1 is also deregulated following administration of HFD in our experiment. Thus, the tissue-specific VNN1 KO mice will also be produced to confirm our results. Third, in terms of our current experimental conditions, it is difficult to isolate the contribution of LV-VNN1 versus endogenous VNN1 in this mechanism. In our research, we measured VNN1 protein expression levels in various tissues of C57BL/6 mice with an HFD. To ascertain the effect of VNN1 on atherosclerosis and exclude the effect of endogenous VNN1, apoE^−/−^ mice were fed an HFD throughout the experiment. A more advanced experimental technique is need to check the expression of endogenous versus transduced VNN1 in vivo.
